# The Epidemiology of Migraine Headache in Arab Countries: A Systematic Review

**DOI:** 10.1155/2020/4790254

**Published:** 2020-06-16

**Authors:** Ashraf El-Metwally, Paivi Toivola, Khalid AlAhmary, Salwa Bahkali, Ali AlKhathaami, Shatha A. Al Ammar, Ibrahim M. Altamimi, Saleh M. Alosaimi, Munazza Jawed, Sami Almustanyir

**Affiliations:** ^1^College of Public Health and Health Informatics, King Saud Bin Abdulaziz University for Health Sciences, Riyadh, Saudi Arabia; ^2^King Abdullah Specialist Children's Hospital, King Abdulaziz Medical City, Riyadh, Saudi Arabia; ^3^Princess Nourah Bint Abdulrahman University, King Abdullah Bin AbdulAziz University Hospital, Riyadh, Saudi Arabia; ^4^King Abdulaziz Medical City, National Guard Health Affairs, College of Medicine, King Saud Bin Abdulaziz University for Health Sciences, Riyadh, Saudi Arabia; ^5^King Abdullah Bin Abdulaziz University Hospital, Riyadh, Saudi Arabia; ^6^King Abdulaziz Medical City, National Guard Health Affairs, King Saud Bin Abdulaziz University for Health Sciences, Riyadh, Saudi Arabia; ^7^Dow University of Health Sciences, Karachi, Pakistan; ^8^Ministry of Health, Riyadh, Saudi Arabia

## Abstract

**Background:**

Recurring migraine disorders are a common medical problem, standing among the top causes of disability and sufferings. This study aimed to evaluate epidemiological evidence to report updated estimates on prevalence, risk factors, and associated comorbidities of migraine headache in the Arab countries. *Design and Setting*. A systematic review was conducted at the College of Public Health and Health Informatics, Riyadh, Saudi Arabia.

**Methods:**

A systematic search in electronic databases, such as PubMed and Embase, as well as manual searches with cross-referencing was performed from 1990 up to 2019. Overall, 23 included papers were rated independently by two reviewers. Studies were eligible for inclusion only if they investigated migraine headache epidemiology in any Arab country and were published in English.

**Results:**

Migraine prevalence among the general population ranged between 2.6% and 32%. The estimated prevalence of migraine headache among medical university students ranged between 12.2% and 27.9% and between 7.1% and 13.7% in schoolchildren (6 to 18 years). Females were found more likely to have migraine than males. The duration of migraine attacks became shorter with increasing age, while chronic (daily) migraine showed increasing prevalence with age. The most commonly reported comorbidities with migraine included anxiety, hypertension, irritable bowel syndrome, and depression. Most common headache-triggering factors included stress, fatigue, sleep disturbances, prolonged exposure to excessive sunlight or heat, and hunger.

**Conclusion:**

The prevalence and risk factors of migraine headache in Arab countries are comparable to reports from western countries. Longitudinal studies are still needed to investigate the prognosis and predictors of chronicity in the arab countries.

## 1. Introduction

Headaches are extremely common and can be defined as a disabling condition that may result in a lower quality of life and disturbed job performance, ultimately creating a significant economic burden on societies [1]. As per the World Health Organization (WHO), half of the adult population worldwide is affected by headaches. These include tension-type headaches, migraines, and cluster headaches. Almost one-third of headache cases in adults are migraines [2]. Migraine is a neurovascular disorder characterized by persistent headache ranging from moderate to severe pain. Typically, it affects only one side of the head, as a pulsating pain, and lasts from hours to days. Its attack usually begins unexpectedly, reaches its maximum in one or more hours, and lasts up to 12 hours [3]. It is also observed to run in families, so it is recognized to have a strong genetic substrate [4].

Various international studies have demonstrated the epidemiology and occurrence of migraine. Global Burden of Disease (GBD) 2013 data showed that it was the 6th leading cause of disabilities around the world and affected more than 10% of the world's population [5]. A systematic review involving 302 community-based studies found that the global prevalence of migraine was 11.6%; i.e., one in ten people suffered from migraine headaches worldwide, of which 16.4% were in Central and South America, 11.4% in Europe, 10.4% in Africa, 10.1% in Asia, and 9.7% in North America. The review also reported that the prevalence was 13.8% females and 6.9% males. Around 12.4% of school and college students were also found to have been affected by migraine, which demonstrates its rising prevalence [6]. Another review presented that approximately 15% of the population gets migraine headaches during the formative and productive era of their lives, usually between 22 and 55 years of age [7].

It was also noticed that if migraine attacks are prevalent in both parents, the risk of descendant disease ranges from 60% to 90%, whereas if the migraine attacks are prevalent among mothers only, the risk of the disease is 72%. In case of prevalence among just fathers, it is around 30% [8]. It has been indicated that if a person has migraine, their mother has 4 times more chances of having a history of migraine than their father [9]. A study confirmed that a higher prevalence of migraine headache among a population leads to various potential socioeconomic damages associated with the treatment and diagnosis [10].

It is known to be a disabling ailment usually coexisting with various morbidities, including neurological disorders (fibromyalgia [11], epilepsy [12], stroke [13], and multiple sclerosis [14]) and psychiatric disorders (panic disorders [15], depression [16], and posttraumatic stress disorder [17]). The major risk factors associated with migraines include stress, anxiety, exposure to sun, sleeping disorders, unhealthy eating habits, smoking, fatigue, and a low socioeconomical level [18]. A systematic review revealed that migraine not only affects the individuals and their families but also reduces their quality of life and social activities [19].

Various studies that reported on migraine, its epidemiology, and other aspects have also come out from the Arab countries; however, pooling of these findings has not been sufficiently achieved as yet. Therefore, we aimed to evaluate the epidemiological evidence in the literature to offer updated estimates on prevalence, risk factors, and associated comorbidities of migraine headache in Arab countries.

## 2. Materials and Methods

We used a systematic review methodology, using the PRISMA guidelines [20], which aimed to establish, through the available literature, the epidemiology of migraine headache in the Arab countries. Electronic searches on PubMed and Embase were conducted over the data from 1990 to 2019 in order to extract the potentially relevant articles. An additional search was done by searching the local journals and bibliographies of the relevant articles. Search terms/key words used either alone or in combination, using Boolean operators, included headache, migraine, Arab countries, epidemiology, prevalence, risk, prognosis, incidence, Saudi Arabia, Egypt, Kuwait, Bahrain, Qatar, Oman, Iraq, Syria, Lebanon, Morocco, Algeria, Sudan, Libya, Tunisia, and Jordan.

### 2.1. Inclusion and Exclusion Criteria

1. Qualitative, mixed-method, and quantitative studies were included.
2. Primary research studies relating to the epidemiology of migraine headache in the Arab regions were included.
3. Studies evidently stating their aims, objectives, and methods were included.
4. Empirical studies in English language published between 1990 and 2019 in peer-reviewed journals were included.
5. Nonempirical studies based on personal opinion, case reports, conference papers, dissertations, and commentary were excluded.
6. Studies conducted in non-Arab countries were also excluded.

### 2.2. Study Selection and Data Analysis

**Figure 1:**
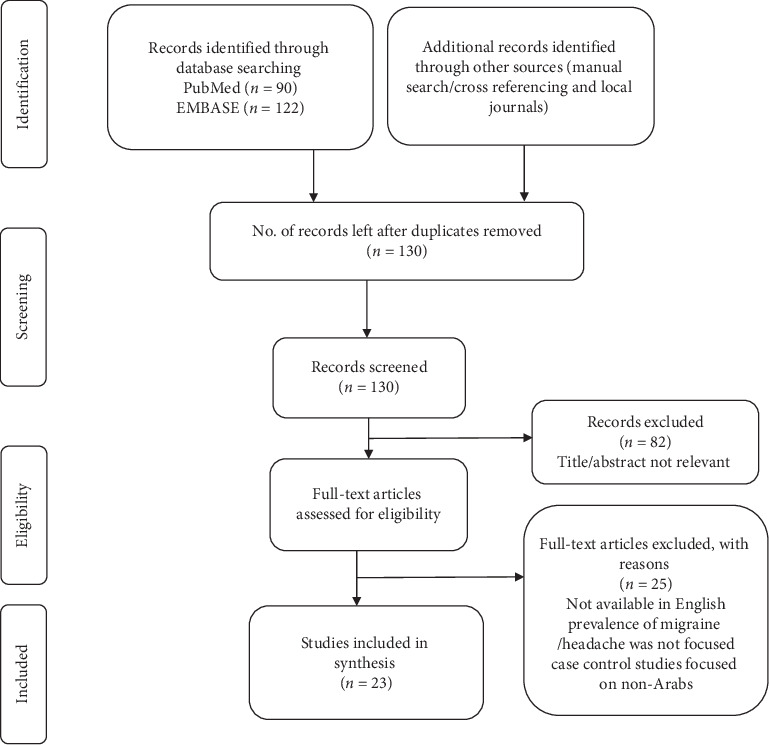
Flow diagram of the included studies.

The abstracts and titles of each identified article were screened independently by two investigators for possible inclusion. Any disagreements were resolved through mutual discussions to reach a consensus. The search conducted electronically identified 90 articles from PubMed and 122 from Embase database. After removing duplicates, the two investigators independently screened and excluded 82 studies due to irrelevance to our research agenda. Overall, 48 of the retrieved articles matched our research topic for review, which were then subsequently evaluated for eligibility to meet our inclusion criteria. After individually reviewing the full text of each study to determine whether the paper should be included or not, 25 more studies were excluded. A total of 23 articles were included in our review (see Figure 1). Using a data extraction table, the data from the included articles were extracted. The following data were collected: author, study duration, year of study, source, study geographical location, study setting, study design, sample size, response rate, diagnostic criteria, and the prevalence of migraines.

### 2.3. Quality Assessment

The quality of the included studies was assessed using the Newcastle-Ottawa Scale (NOS) [21]. A modified version for cross-sectional studies was adopted, which had also previously been used in different published studies [22, 23]. The scoring of the modified NOS ranges between 0 and 5: unsatisfactory studies receive NOS scores of 0–2, satisfactory studies receive an NOS score of 3, good studies receive a score of 4, and very good studies receive 5/6 NOS scores.

## 3. Results

### 3.1. Studies Characteristics

Table 1 gives a detailed description of the relevant extracted data from the 23 qualifying epidemiological studies, including prevalence/risk factors of migraine headaches. All of the 23 articles used reliable methods of data collection such as questionnaires and were population-based studies with prevalence data for migraines. Eight studies were conducted in Saudi Arabia, three in Egypt, three in Kuwait, two in Jordan, two in Oman, two in Qatar, and one study each in Tunisia, United Arab Emirates, and Yemen.

Thirteen studies used the International Headache Society's (IHS) criteria for the diagnosis of migraine, while the remaining studies used the WHO & International Classification of Diseases (ICD), ICD-II criteria, ICD-IIIb, and Identification of Migraine (ID Migraine™). Sample size ranged from 222 to >33,000. Overall response rates in the study samples ranged from 76% to 99%, while eleven studies failed to report any response rates. None of the research papers in mixed populations involved subjects who were younger than 6 years old, and most of the participants were at least 18 years old.

In general, all the selected studies were cross sectional and their appraisal score is shown in Table 1. Most studies (7) qualified as very good quality studies with scores 5-6, five studies were classified as good studies, and seven studies were found to be satisfactory, while only four out of twenty-three fell in the unsatisfactory range.

### 3.2. Prevalence of Migraine

None of the research papers presented data on the incidence of migraines; only prevalence estimates were reported. Migraine prevalence among the general population was estimated in ten articles [24, 26, 32–35, 38, 40, 42, 43] and showed a range between 2.6% and 32%, while four other studies [27, 30, 36] were performed on clinic attendees and showed a prevalence ranging from 7.9% to 78.5%. Five other studies [9, 10, 25, 37, 41] indicated that the prevalence of migraines among school children (aged 6 to 18) ranged from 7.1% to 13.7%. Three studies also estimated prevalence among medical university students [28, 31, 39] to reveal a range of 12.2% to 27.9%. In addition to these, there was one study by Garah et al. [29], which concluded that the prevalence of migraine among exclusively female university students was as high as 61.77%. Six other studies [27, 28, 33–35, 39] also indicated that migraines were more prevalent among females, whereas one article by Jumah [26] contrastingly reported a higher prevalence among males. The duration of migraine attacks was observed to have become shorter with increasing age, while chronic (daily) migraine showed an increasing prevalence with age [35]. Moreover, headaches were more prevalent among patients with analgesic overuse [40].

### 3.3. Risk Factors and Comorbidities of Migraine

The burden of migraines strongly increases according to its linkage with other neurological, psychiatric, cerebrovascular, and cardiovascular diseases. Migraine is associated positively with a myriad of disorders. This was also confirmed by Kandil et al. [35] in a study that indicated that the most common comorbidities with migraines were hypertension, anxiety, irritable bowel syndrome, and depression. Delineating migraine comorbidities is essential as it can aid in improving treatment approaches and help to understand the possible pathophysiology of migraine. Many other studies [8, 9, 28, 29, 32, 36] investigated the most common headache-triggering factors among participants, and these included stress, fatigue, sleep disturbances, prolonged exposure to excessive sunlight or heat, and hunger.

## 4. Discussion

Studies reviewed in this article revealed variable results. Prevalence and epidemiology of migraine headache varies in the Arab countries; however, the range of prevalence remains within the estimated global range. The factors that were found to trigger migraine included stress, sleep disturbances, hunger, fatigue, loudness, certain smells, prolonged exposure to excessive heat or sunlight, and family history of migraine. The relieving factors identified were medications, darkening, massage, rest, and sufficient sleep. Since the studies included participants from almost all age groups, the age range for the highest risk of migraine could not be estimated. However, females after their fourth decade of life were found to be more prone to develop migraine than males. Additionally, few comorbid conditions like anxiety, depression, hypertension, and irritable bowel syndrome were also found to be significantly associated with migraine.

As stated earlier, the prevalence of migraines varied among different Arab countries. For instance, the rates of migraine in Saudi Arabia were quite high compared with Oman and Qatar, regardless of the fact that they share the same culture, economics, ethnicity, and climate [26, 36, 38]. The epidemiological studies of migraines are rather difficult due to the uncertainty of its clinical criteria as well as nonrandomized selection of groups of subjects [45]. Recent population-based studies from western countries demonstrated the prevalence of migraines to be 10% to 12% [15], while in European studies, it has been reported to be within a range of 12%–28% [46]. Some other studies did however find lower migraine prevalence in Asian (1%–22%) and African populations compared with European (10%–25%) and American countries (9%–16%) [47].

Young women remain the most vulnerable population for migraines and headaches in the Arab countries. A Korean study by Kim et al. reported that women were three times more prone (9.2%) to migraines than men (2.9%) [48]; these findings are in line with our review. On a similar note, women in western countries were also found to be two to three times more prone to migraines [49]. Female predominance has also been noticed in various other populations as well [50]. It is also notable that migraines have been reported to be at their worst between the age of 30 and 40 years; these findings are similar to the estimated age-related prevalence in Asian countries [49]. Its highest level is observed during the most productive years of life, i.e., from 25 to 55 years, while 90% of migraine sufferers endure their first attack before the age of 40 [30]. The age of participants in the selected studies varied as some included children and adults alike, while some were conducted only for school-going children and adolescents.

Risk factors for migraines were identified in this review. Likewise, many other studies also noticed certain factors that alter the occurrence of migraine. For instance, Bigal and Lipton proposed age, low education and socioeconomic conditions, head injury, obesity, stressful occasions, and the overuse of caffeine and medications to be factors affecting migraine headaches [51]. Similarly, in Japan, lack of sleep, mental stress, and fatigue were the main headache triggers [52]. As for our review, various studies noticed frequent migraine attacks in women to be associated with the highest risk for developing depression [53, 54]. Linstra et al. reported an increased risk of cardiovascular events including stroke and cardiac ischemia particularly in women having a history of migraines [55]. Recent reports have also demonstrated an association of migraines with several gastrointestinal disorders, including irritable bowel syndrome [56]. Hence, previous literature incorporated many of the findings that were picked up by the present review.

### 4.1. Assessment of Possible Biases of Included Studies

In this systematic review, the objective and inclusion criteria of studies were evident. Steps were taken to minimize the possible bias by reviewing, validating, and data extraction, and it was done independently by two investigators. Selection and information biases are common in systematic review of cross-sectional studies. One possible bias was publication bias that we included studies that were published in English. Second, since we did not include any unpublished research or so-called “grey literature,” publication bias was not excluded as well. We did not include studies that conducted before the year 1990 and those published after the end of 2019, so there exists a possibility that at the time of publication, it might not include the latest prevalence. However, we tried to overcome identification bias by searching for the literature electronically (two databases) as well as manually. Moreover, some studies have been published in the early nineties leading to unavoidable heterogeneity in population characteristics, methods, and interventions. Another bias of the included studies is self-selection bias that might be attributable to smaller sample sizes and no description of nonrespondents. Bias assessment is different from quality assessment; therefore, studies with a higher score in quality assessment with larger sample size, appropriate methodology, and used validated diagnostic criteria for migraines have a low risk of bias.

### 4.2. Methodological Quality Appraisal

The quality of the included studies assessed in this systematic review is a matter of concern because this can lead to biases and, in some cases, inaccurate estimates (either under or overestimation of actual prevalence). We used the Newcastle-Ottawa Scale (NOS) for quality assessment of cross-sectional studies. Overall, few good quality studies about migraine headaches were found mostly from Arab countries. The low scores of quality appraisal are due to inefficiencies in methods and reporting of the studies that included in the present review. The majority of the studies with unsatisfactory scores had smaller sample sizes and no description of nonrespondents, which might be due to self-selection bias. Most of the selected studies used multistage sampling and door-to-door surveys. Multistage sampling methods lead to an underestimation of the true prevalence of migraine, while an overestimation of precision. It can also result in high numbers of loss to follow-up or nonresponse rate that occur from screening to diagnostic assessment time [57]. Also, some studies did not use validated tools for the measurement of migraines. Moreover, in few studies, method of outcome evaluation was not appropriate. For instance, in some unsatisfactory studies, methods used to assess outcomes were not described. Few studies used adjustment of the confounder and regression analysis while assessing outcome, hence yielding high scores in the quality appraisal.

### 4.3. Strengths and Limitations of the Study

The present review possesses a few limitations. Since the included studies utilized different age groups, a thorough idea about the most common age group for the occurrence of migraine could not be identified. The studies also had methodological differences that would have influenced the results of the review. The review suggests striking differences in the prevalence of headache among Omani and the Saudi populations. These differences can be linked with the study setting and design, such as studies involved in door-to-door, community-based, school-based, and medical-student-based surveys. However, despite various studies from the Arab countries that estimated the prevalence and other modifying factors for migraine, lesser work had been done for combining those findings. Although the included studies provide pieces of evidence about the predicting factors, prevalence, course, and diminution of migraine, we did not find studies about the incidence and lifetime manifestation of migraine in Arab countries. This might be due to the inclusion and exclusion criteria of study selection. The major strength of this review is the attempt to gather these studies from various Arab countries and analyze them systematically to explore important factors affecting migraine headaches. All the selected studies' characteristics were summarized in a table. Additionally, this study allowed us to suggest a recommendation for the direction of future epidemiological studies.

## 5. Conclusion

Despite being one of the most disabling headaches, migraine is still underdiagnosed and undertreated. It is unequally distributed among people of mental and physical work, different socioeconomic levels, and residents of the city and the urban regions. Earlier, it was believed that more educated people and urban dwellers were more likely to suffer from migraine. However, contemporary studies have shown that this pattern can be traced only in the population of patients seeking treatment. It turns out that more educated patients are more worried about their headaches and are more likely to consult a doctor. In the general population of patients, these differences are not preserved. Migraine was found to be more widespread in women than men as well as more predominant in the urban population. Furthermore, it potentially influences the daily life activities of the patients, including social occasions, employment, and schooling. This forms a challenge not only for the patients but also for physicians with respect to appropriate recognition, prevention, and timely treatment. Therefore, longitudinal studies are needed in the future in investigating the prognosis and predictors of chronicity in the Arab countries to retrieve more accurate results.

### 5.1. Recommendations

Since migraine causes a significant decrease in quality of life, it should be addressed in an adequate manner as well. According to the National Outpatient Care Service of the United States, around 10 million people visit doctors for headaches per year [58] and many medications are prescribed. About AED 400 million is spent by the Arab population on prescription pain relievers for headaches annually [38], which is an alarmingly high figure. By conducting reviews like this one, findings from within the country as well as neighboring countries can be pooled in order to identify common epidemiological risk and relieving factors. Additionally, well-designed epidemiological studies involving Arab nations will further aid in learning more about this condition. Appropriate preventive strategies at the level of patients and physicians should also be considered in primary settings. It is well known that females are more prone to migraines than men; therefore, this vulnerable segment of the population should be targeted for such interventions. Clinicians, especially general practitioners, should aspire not to just relieve the current pain and disability but to also avoid its progression along with a focus on decreasing attack frequency, avoiding overuse of medication, prescribing preventive drugs, encouraging behavioral therapies, and preventing complications with an eye on the patient's comorbid conditions. These should all be a part of migraine therapy to reduce its burden and improve the overall quality of life of the sufferers.

## Figures and Tables

**Table 1:** Characteristics of included articles.

Author [ref]	Country	Study duration	Study population	Study type	Diagnostic criteria	Sample size	Study quality score (8)	Migraine Prevalence (%)	Other results
Abduljabbar et al. [24]	Saudi Arabia	Oct 1994–Mar 1995 (6 months)	>15 years old	A community survey (door-to-door)	IHS criteria	5,891,473 suffered from headache	3	2.6	Most of headache sufferers were women. The age-specific rate was increasing with a peak in the sixth decade.

Ai‐Rajeh et al. [8]	Saudi Arabia	Dec 1983–Nov 1988	All population	Evaluation of patients with headache as the key reason for their visits in the hospital.	The definition of Blau, 1984.	222	1	22	Migraine showed a higher prevalence among Saudi females only in the fourth decade (female-to-male ratio of 4 : 1). 10% of the cases presented positive family history for headache. Major headache precipitating factors were stress, prolonged exposure to excessive sunlight or heat, and hunger.

Jumah et al. [25]	Saudi Arabia	1 year	Schoolchildren (6–18 years)	A cross-sectional, questionnaire-based study	IHS criteria	1,400	3	7.1	For migraine, in both boys and girls, prevalence rate sharply increased from around 2% to around 9% at age 10 to 11.

Jumah et al. [26]	Saudi Arabia	1 year	18–65 years	A country-wide population-based cross-sectional survey		2,421	4	32	This study shows a high prevalence of migraines among men.

Almalki et al. [27]	Saudi Arabia	—	General population	A cross-sectional survey study	IHS criteria	354	2	78.5	Migraine prevalence was found to be higher in urban areas and among females. An association was found between migraines and high rates of unemployment. The most common symptom associated with migraine was nausea.

Al-Tulaihi et al. [10]	Saudi Arabia	2002-2003	High school students (16–21 years)	A cross-sectional, questionnaire-based study	IHS criteria	1750	3	7.7	Migraine shows higher prevalence in females than males.

Ibrahim et al. [28]	Saudi Arabia	2014-2015	Medical students (2nd–6th year)	A cross-sectional study	ID Migraine test™, Numeric Pain Rating Scale (NPRS).	566	6	26.3	The major migraine predictors were found to be functional gastrointestinal disorders (FGIDs), family history of migraine, female gender, and enrolment in the second academic year. The most common triggers were exam stress and sleep disturbances.

Garah et al. [29]	Saudi Arabia	2013-2014	Female university students	A cross-sectional study	IHS criteria	395	5	61.77	The most common triggers were physical stimulation (like light, loudness, change in weather, and certain smells). Among students, the most important migraine relieving factors were rest and sleep (63.5%), medication (33.2%), followed by darkening room (30.3%), massage (20.1%), and coffee drinking (18%). A significant association was found between migraine headache and family history of migraine and studying in theoretical colleges.

Jamal et al. [30]	Kuwait	Mar 2003–Jun 2003	15–80 years	A cross-sectional study	IHS criteria	290	1	11.7	There was no evidence to prove a positive correlation between high blood pressure and headache.

Al-Hashel et al. [31]	Kuwait	2012-2013	Medical students	A cross-sectional, questionnaire-based study	Identification of migraine (ID Migraine™)	621	2	27.9	The most common triggering factors in students were stress (24.9%), irregular sleep (20.8%), and substantial reading tasks (18.5%). Migraine prevalence showed an increase in the final two years of education.

Al-Hashel et al. [32]	Kuwait	Jan 2016–Apr 2016 (4 months)	18–65 years	A population-based cross-sectional survey (door-to-door)	ICD-II criteria	15,523	4	23.11	Highly prevalent condition having a significant impact on daily living activity, employment/schooling, and social occasions of patients.

Badry et al. [33]	Egypt	Jul 2009–Jan 2012 (31 months)	>8 year old	A door-to-door screening and an examination survey	WHO	33,283	5	2.8	The highest prevalence was recorded in the elderly population (60+ years, 8.0%) and among the age group 18–39 years (5.4%).

El-Sherbiny et al. [34]	Egypt	Jan 2014–Oct 2014	15–83 years	A community-based, cross-sectional observational descriptive survey.	ICD-IIIb	2600	6	17.3	Migraine shows higher prevalence in females than males.

Kandil et al. [35]	Egypt		All population	A cross-sectional population-based study (door-to-door)	HIS & ICD-II	4,700	3	10.55	Chronic or daily migraine was more common in females (35.3% versus 20.7% for males). Migraine attack duration was found to get shorter with increasing age, but the chronic (daily) migraine showed an increasing prevalence with age. The most common comorbidities with migraines were hypertension, anxiety, irritable bowel syndrome, and depression.

Bener [36]	Qatar	Oct 2004–Dec 2004	>15 years old	A cross-sectional population-based study.	IHS criteria	913	4	7.9	Most common warning symptoms before headaches were weakness (30.4%) and abnormal vision (53.0%). Factors that were found to make headache worse included stress (71.8%) and weather (49.5%).

Bessisso et al. [37]	Qatar	Mar 2001–Apr 2003	Schoolchildren (6–17 years)	A cross-sectional survey	IHS criteria	851	5	11.9	The most common triggers were found to be lack of sleep (17.6%) and fatigue (35.8%).

Deleu et al. [38]	Oman	1999-2000 (2 year)	>10 years old	A community-based study	IHS criteria	1,158	3	10.1	In this study, migraine prevalence did not show significant gender difference (4.5% in male and 5.6% in female).

Deleu et al. [39]	Oman	2001	Medical students	A college-based, cross-sectional survey	IHS criteria	403	4	12.2	A significant gender distribution difference of migraine prevalence was 6.6% of men and 15.5% of women.

Alzoubi et al. [40]	Jordan	Jan 2007–Nov 2008	18–85 year	A community-based, cross-sectional study	—	4,836	3	7.7	Headache and overuse of analgesics was prevalent.

ALBashtawy et al. [41]	Jordan	3 weeks	School students (16–18 years)	A school-based, cross-sectional study	IHS criteria	754	4	8.8	This study indicated that migraine prevalence was initially high and increased with age.

Romdhane et al. [42]	Tunisia	Jul 1985		A full-scale survey evaluated by a second survey.	WHO & ICD	1,673	5	8.6%	Migraine prevalence ratios in Nigeria, Ecuador, and Kelibia were equivalent.

Abdo et al. [43]	Yemen	Jul 2010–Sep 2011	18–85 years	A cross-sectional observational study	IHS criteria	12,640	3	14.48	The study demonstrated a high prevalence of headache in Kuwait with 76.5% of the subjects experiencing headache attacks at least once per year

Bener et al. [9]	United Arab Emirates	Oct 1995–Jun 1996	Schoolchildren (6–14 years)	A cross-sectional population-based study	IHS criteria	1,159	5	13.7	Headache prevalence increases with age. Highest rate was found among 13-year-olds (17.5%).

Sabra et al. [44]	Saudi Arabia	2015	General population	A cross-sectional population-based study	IHS criteria	1002	4	10.8%	Out of the atypical complaints, 86% of the patients had a history of concomitant typical presentation.
